# ‘Bottom dog men’: Disability, Social Welfare and Advocacy in the Scottish Coalfields in the Interwar Years, 1918–1939

**DOI:** 10.3366/shr.2017.0335

**Published:** 2017-09

**Authors:** Angela Turner, Arthur McIvor

**Affiliations:** ^A^ University of Strathclyde; ^B^ University of Strathclyde

## Abstract

This article connects with and builds on recent research on workmen's compensation and disability focussing on the Scottish coalfields between the wars. It draws upon a range of primary sources including coal company accident books, court cases and trade union records to analyse efforts to define and redefine disability, examining the language deployed and the agency of workers and their advocates. It is argued here that the workmen's compensation system associated disability with restricted functionality relating to work tasks and work environments. Disability became more visible and more closely monitored and this was a notably contested and adversarial terrain in Scotland in the Depression, where employers, workers and their collective organisations increasingly deployed medical expertise to support their cases regarding working and disabled bodies. In Scotland, the miners' trade unions emerged as key advocates for the disabled.

Coal mining communities had the highest proportion of disabled people than any others before the Second World War because of the chronic diseases and high injury rate that characterised miners' work.^2^ Mining disasters with large numbers of fatalities are well known, but these communities were also characterised by a steady flow of damaged bodies, with large numbers who had lost limbs, were paraplegics, had brain damage and other forms of mental health disabilities and had varying degrees of eyesight deficiency (nystagmus), arthritis, leg and arm joint damage (‘beat knee’; ‘beat hand’) and respiratory disability, including pneumoconiotics. It was estimated in the 1920s that over an average working life of forty years more than a half of all British miners would suffer from a serious disabling injury or contract a chronic life-limiting industrial disease, whilst additionally each miner was liable to suffer on average seven minor disabling ‘accidents’ necessitating more than a week's loss of work.^3^ In the 1930s, 80% of all workers in the UK who were classified as long-term disabled (defined as over ten years) by work-related injuries and diseases, were in the mining sector.^4^

From 1897 the social welfare system designed to monitor and address the consequences of this industrial carnage was the Workmen's Compensation Act (hereafter WCA). This emerged from recognition that existing provision through the Poor Law, workers' mutual insurance agencies, savings funds and friendly societies, charity and the Employers' Liability Act (1880) was inadequate and that employers needed to accept direct statutory responsibility for damaged, disabled and killed workers. Studies of workmen's compensation have tended to focus on the formulation and operation of policy at the national level and international comparisons.^5^ This article engages with this literature through the lens of Scottish experience between the two world wars, 1918–1939. Recent research has to some degree refocused attention on the specificities of the local context (including cultural dimensions) and the competing discourses of the key players around work-related disease, especially related to miners' respiratory disability, with a notable focus on South Wales.^6^ This article builds on and connects with such work by focussing attention on injury and disability in the Scottish coalfields, exploring the efforts to define and redefine disability that went along with the workmen's compensation system and examining the language deployed and the agency of workers and their advocates in what might be described as an injured or disabled workers' movement in the interwar years.^7^ It is argued here that this was a notably contested and adversarial terrain in Scotland, where employers, workers and their collective organisations increasingly deployed medical expertise to support their cases regarding working and disabled bodies.

Workmen's compensation as an element of the emerging welfare state has been surprisingly neglected in Scotland. Studies of poverty, health and welfare have overlooked work injuries and disease as a cause of deprivation and focussed attention on the Poor Law.^8^ Scottish miners have been extensively studied, but research has tended to focus on the workplace and industrial politics of these workers and their collective organisations. Alan Campbell's seminal studies, for example, almost completely neglect disability and the body, occupational health and safety and issues around miners' welfare and workmen's compensation for injuries and disease.^9^ A partial exception is Leah Leneman who investigated workmens' compensation claims in the Wemyss Coal Company in Scotland between 1906 and 1924.^10^ Whilst Leneman's company case-study approach raises important questions about the local and regional differences in compensation cases and suggests a more nuanced picture of the application of the legislation on the ground than wider national studies, her work does not explicitly focus on disability.

Both Brendan Gleeson and Anne Borsay's work in the field of disability history established a theoretical framework for the study of such disabled workers through the lens of materialism.^11^ Gleeson's work in particular notes the ways in which increasing mechanisation in production created and standardised ‘normal’ worker's bodies at the expense of the unfit or disabled.^12^ However the military disabled have received more attention than those disabled by their employment.^13^ Julie Anderson, however, does point to the significance of common conditions amongst coal miners, such as the repetitive injuries of ‘beat’ hand, knee or elbow (bursitis) as well as high levels of pneumoconiosis.^14^ Similarly Joanna Bourke discusses the significance of disability in coal mining and points to the excessive levels of risk whilst Noelle Whiteside and others have highlighted the high levels of compensation claims in coal mining where ‘incapacity and sickness was evidently endemic’.^15^ Other important work, including that of Julia Moses, has focused on defining accidents and risk and exploring the contested nature of liability and compensation, including international pressures on regulatory policy, especially in the period 1884–1920.^16^ However, in all these accounts discourses of disability and the lived experience of disabled miners and their *agency* in compensation struggles in the context of the interwar Depression are not examined in anything other than a cursory fashion.

Mine workers were not passive victims but active agents in constructing a particular discourse around the body at work and in the injury and disability compensation struggles that dominated the industry from the late 1890s through to the Second World War. A key player in this was the miners' trade unions. Research on the history of occupational health and safety has been divided on the role of the trade unions with one interpretation defining the unions as ineffective and strategically failing to prioritise protection of the body at work (placing job security and wage maintenance above health and safety), whilst at the other extreme unions are perceived as pivotal interlocutors. Bloor, Melling and Bufton (in relation to silicosis), McIvor and Johnston (in relation to coal workers' pneumoconiosis) and Long (in relation to the TUC) have done much to rehabilitate the unions in this story whilst elaborating on the language deployed and the complexity of such relationships.^17^ That said, the role of the trade unions as advocates for disabled workers and the specific Scottish experience of disability politics in mining (and more widely) has been neglected and merits attention in its own right, not least because of the peculiarities and specificities of Scottish culture and institutions through which workmen's compensation policy was refracted. The latter includes a more markedly confrontational industrial relations system where anti-trade unionism was more entrenched within industry and, partly in reaction, workers' class consciousness was sharper and the labour movement more overtly political and to the left. This is perhaps most manifest in the first quarter of the twentieth century and in the (albeit contested and controversial) phenomena of ‘Red Clydeside’.

The reality was somewhat less prosaic and recent studies have refined both the idea of autocratic and intransigent management and dismissed the more exaggerated claims about the reach of socialist politics, communist influence and revolutionary ferment on Clydeside and the coalfields. Scottish employers' were not uniformly autocratic and strategies ranged across a spectrum, whilst workers were divided and characterised by a multiplicity of identities. Nonetheless, the politics of workmen's compensation was confrontational and mining workers, through their trade unions, were capable of challenging dominant managerial, state and medical discourses and understandings.

The First World War represented a real challenge to the progress that had been made regarding workmen's compensation in that the wartime intensification of work associated with the munitions production drive (and need for coal as a fuel in industry) led to more accident injuries, hence swelling the numbers of disabled people in mining communities. Wartime pressures to ‘perform’ patriotic masculinity, to ‘act as men’ and support the war effort also led to taking more risks and some injured and disabled miners returning to work prematurely before they were fully fit.^18^ Men disabled by their work were joined by injured and disabled soldiers who returned back to their mining communities. These men and their families were hit hard by rampant wartime inflation (the cost of living roughly doubled over 1914–20). The hardship of disabled people was particularly acute because they failed to share fully in the collectively bargained wage rises for coal mining employees in the 1910s.^19^ The disabled community were also particularly disadvantaged in the fight for jobs during the interwar Depression, not least in mining communities where mass unemployment became the norm in the 1920s and 1930s.

Our research examines the workmen's compensation system as it operated in Scotland, exploring how it allowed miners to claim against their employers for their injuries and resulting disablement in new ways (including for a number of defined chronic occupational disabilities). This experience needs to be understood within the specific context of the Scottish coalfields, where there was a particularly entrenched anti-trade union and authoritarian managerial culture, more advanced levels of coal mine mechanisation than elsewhere and a prevailing high risk *macho* work culture.^20^ What follows reveals the disputed terrain of disability compensation struggles in Scotland and the ways in which the miners, employers and medical professionals framed narratives of disability and bodily damage that strengthened their arguments for and against monetary awards.

## Workmen's compensation: Redefining disability

Replacing the ambiguous and highly contested notion of ‘employer liability’ the Workmen's Compensation Act passed in the UK in 1897 provided for the first time (on paper at least) a statutory no-fault system of compensation for those injured, disabled or killed as a result of ‘accidents’ at work.^21^ It recognised, as Moses has argued, a distinct ‘foreseeable and calculable’ occupational risk.^22^ Under the 1897 WCA employers were made directly responsible unless they could prove the ‘wilful negligence’ of others (for example, by workers) and financial payments were directly made by the employers to workers with most insuring themselves against this risk. In some cases industry employers' associations took on this insurance responsibility.^23^ A decade later (1906) the system was extended to cover six common industrial diseases (including anthrax, mercury and phosphorus poisoning)—and the list of such ‘prescribed diseases’ was added to periodically thereafter on recommendations from a committee of experts (the Industrial Injuries Advisory Committee). There were over 30 such disabling diseases recognised for compensation purposes by the late 1930s, including, in coal mining, nystagmus, beat hand, wrist and knee, and silicosis.^24^ Injury and disease was assessed by medical examinations and ‘expert’ medical referees, thus bringing medical surveillance to the workplace and subjecting workers' bodies to unprecedented scrutiny. Actual compensation levels, however, were limited and low, set at 50% of average earnings over the year prior to the injury and only applying after a two week waiting period—a clause designed to minimise ‘malingering’.^25^ Contested cases could be taken to court where, in Scotland, a Sheriff adjudicated. Outside of wartime, the Workmen's Compensation system became the primary conduit through which the state, employers and medicine interacted with injured and disabled people prior to the Disabled Persons Act of 1944. It marked a paradigm shift in social provision for one significant group of disabled people who previously were almost entirely dependent upon the Poor Law, charity and family support.

The Scottish evidence elucidates how the meanings of categories such as ‘able bodied’ and ‘disabled’ were continually contested through cases considered under the WCA. Whilst miners' impairments and disabling bodily injuries are often described in detail in workmen's compensation cases, they are at the same time also described as ‘fit’ or ‘in good health’ and ready to come off compensation payments. For example during First World War one Fife miner was classified by the medical referee as fit to ‘resume his work underground as practically a one-eyed miner’.^26^ Another, who in 1928 had sustained an injury to his left hand and was left with substantial weakness and impairment of function, was described as ‘at present for practical purposes a one handed man’.^27^ He was therefore classified as ‘fit for work mainly performed by the right [hand], the left one assisting’.^28^

Whilst the physical limitations and alleged recovery of disabled miners may be referenced, often the miner was tasked with adapting to his new circumstances and changes in his body or to find work in suitable environments. For example, a wartime miner who had sustained substantial injuries was described as ‘fit to resume his work as a machineman though it may take time for him to adapt to his altered circumstances and regain his full earning capacity with confidence’.^29^ Another miner, who lost his eye in an accident in 1933, was classified as fit for work below ground as long as there was ‘good head room’ and the job did not involve too much stooping.^30^ Thus the disabled bodies of the men are often considered on the basis of their economic potential. For example in 1926 one miner was said to be ‘disabled from earning full wages’ by miner's nystagmus which is aggravated by darkness and exertion.^31^ His disability lay in the reduction of his earning capacity by partial blindness rather than in the diagnosed impairment or illness. Whilst compensation legislation for disabled ex-servicemen focussed on medical diagnoses and was ‘based on disfigurement rather than loss of earnings’, workmen's compensation rates up to Second World War were calculated on the loss of earning capacity alone and not for the injury or disfigurement itself.^32^ The system in the UK differed significantly from the European continent where loss of function in different body parts determined the level of compensation award—such as a 60% award for a loss of an arm or leg; 25% for the loss of one eye; 10% for up to two fingers.

Historians and disability theorists who have explored interactions between medicine and the disabled body have often focussed on the ways in which medicine marked the disabled body as ‘damaged’ or in need of treatment to regain functioning or to restore the body to aesthetic norms. The interactions involved in workmen's compensation in the UK, however, often involved doctors and medical referees framing disability in a way more in line with a social model type of analysis. Miners' disabled bodies are considered in terms of functionality and suitability for particular tasks. The doctors considered the physical environment they worked in as well as the ways in which they could adapt their ‘mind-set’ in returning to work. The key motivation for this may have been to limit the levels of compensation payments. Nevertheless, disabled miners are often presented as ‘fit’ to remain part of the workforce and to remain productive. Disability was not fixed but was considered in relation to functional ability. Historians such as Anderson therefore have identified the shifting, fluid nature of disability and the ways in which disabling conditions are understood in relation to medical advancements or by ‘different cultural ebbs and flows’.^33^ The limited application of a social model type of approach in interactions with these disabled workers, however, does not seem to go beyond the arena of medical assessments in compensation cases. There is little evidence of attempts to adapt working environments or to consider barriers to these disabled workers. The disabled miner was urged to take responsibility, adapt to their new circumstances and utilise their remaining physical abilities. Borsay has shown how this concentration on bodily limitations in orthopaedic medicine resulted in a focus on ‘personal pathology to which the individual had to adjust’ and failed to acknowledge social or environmental barriers.^34^ Injured miners are thus often judged on their attitude towards their disability or injury. Anderson has identified a similar process in the rehabilitation of disabled soldiers where they were often judged on their ‘will to get well’.^35^ One example of this in a mining compensation case is that of a forty-three year old brusher who is described as being in the ‘better class of workmen’ as he has continued to ‘work up to his capacity’ as a repairer since he suffered from a back and body injury after a fall of stone.^36^

## Malingering and Medicine

On the other hand miners who were deemed to have exaggerated their disabilities or who were felt to be shirking their responsibilities to work were presented as a real threat to the industry through costly unwarranted compensation claims. Moses points to a perceived ‘crisis of malingering’ in civilian life in the early twentieth century linked to the emergence of compensation cultures in Germany, Britain and Italy.^37^ Bourke notes how Workmen's Compensation medical assessors such as Dr Dickson in Fife recorded an increase in recovery times along with the increasing availability of compensation.^38^ Some company-employed doctors denied that certain occupational disabilities made any difference to workers' lives, such as industrial deafness.^39^ Whilst malingering was felt to be on the increase by employers and the Ministry of Health, Bourke notes, however, that the rise in injury compensation claims over 1900–1920 can also be attributed to factors such as ‘speeding up’ of industrial processes with higher levels of mechanisation and associated risk, and that doctors specializing in malingering workmen generating panic about fraudulent cases.^40^ Bartrip has added the point that growing numbers of claimants may well have represented a growing awareness of the legislation rather than any real increase in risk.^41^

Scotland was one of the most highly mechanised coalfields in the UK by the interwar years and Alexander Renfrew has argued that the interwar Depression increased injury risks markedly.^42^ As with the 1980s recession, this was linked to an all-pervasive if uneven managerial drive to remain competitive by cutting costs, reorganising and ‘rationalising’ work systems, increasing work hours and reducing wages. In a payments-by-results system this incentivised overworking to make up earnings in an insecure labour market. Moreover, labour discipline was tightened by the spectre of mass unemployment, the increasing precarity of jobs as pits closed and the growing marginalisation of the trade unions as membership levels (and power) fell sharply after 1920. What is evident (table 1) is that fatalities and injuries did rise in coal mining across the UK in the worst years of the interwar Depression.

**Table 1:** Average annual death and injury rates in UK coalmining, per 100,000 man shifts worked, 1922–36.

	killed	injured
1922–26	0.40	65.1
1927–31	0.43	69.3
1932–36	0.44	65.6

*Source*: *Royal Commission on Safety in Coal Mines*, Report, Cmd 5890 (1938), 65.

In this environment, cases relating to compensation led to increasing levels of conflict between workers, employers, insurers and unions, inside and outside the courts.^43^ Leneman argues that in the case of the Wemyss Coal Company, employers attitudes hardened in the Depression and they were much less likely to accept the workmen's stories as genuine.^44^ Whilst earlier cases mainly report the ‘facts’ of the injury or accident, miners in early 1920s' cases are often ‘alleged’ to have contributed negligently, and in effect injured themselves.^45^ Not only were workers blamed but the workmen's compensation system also continued to encourage employers to identify and dismiss older, partially disabled and more vulnerable workers who were deemed to be too high an insurance risk. An article in the *Times* in 1910 referred to this as the ‘weeding out’ of ‘elderly and delicate men’.^46^

Texts such as *Malingering and its Detection*, written by a physician from the Edinburgh Royal Infirmary and published in 1912, pointed towards ways in which to identify ‘undesirables whose main object in life is to trade upon the generosity of their fellowmen’.^47^ McKendrick argued that with the WCA ‘all injured workmen are tempted to make the most of any injury received while at work’.^48^ In one case involving a worker with pronounced ‘miner's spine’ the injured man was accused by the medical referee of being ‘troublesome’ by involving an agent and in making more of his injuries. This was despite the fact that the curvature of his spine was described as ‘more marked than usual’ and the doctor's opinion was that ‘he is not yet able for full work and may not be for years, if ever’.^49^ The case notes, however, still describe him as ‘a healthy looking man’ who is ‘fit for more that he is doing at present’.^50^ This belief that miners were liable to exaggerate injuries if potential pay-outs were set too high was also expressed by others such as William Fletcher, President of the Mining Association (the coalowners' national organisation) who had argued in 1897 that higher payments ‘would afford a great temptation to magnify small injuries and prolong the period of incapacity’.^51^ He went on to suggest ‘the experience of the Benefit Societies and kindred clubs in all the mining districts prove that the existence of this temptation constitutes a very serious evil’.^52^ Cases of fraud concerning compensation also appear in the press between the wars. For example in 1924 one miner was reported to have been sent to prison for thirty days after having the ‘effrontery’ to work at another pit after reporting himself unfit for work at Kinnell Colliery.^53^ Another in 1926 reported a disabled miner accused of ‘fraud’ when he did not declare his disability war pension.^54^

Bourke has noted that the industrially injured were often treated as if they were ‘on trial’ suggesting that ‘insurance doctors tended to be hostile to workers’.^55^ This was a contested terrain. Indeed trade unions often criticised the use of medical referees paid by coal companies accusing them of bias and called for the employment of full-time state appointed and paid medical referees.^56^ Medical men, such as McKendrick, argued that workmen's own doctors were more prone to bias and ‘being deceived’ whilst company doctors and medical referees were above reproach, suggesting ‘surely no doctor could be accused of selling his soul and conscience for the sum of a few guineas’.^57^ Workers and their representatives argued the opposite: that company doctors were ‘tainted’ and had a propensity to support the employers’ case.^58^ The weight that should be given to medical reports was significant. In a case brought before the House of Lords in 1929 concerning an injured miner and Clyde Coal Company Ltd. It was held that ‘where a certificate given by the medical referee was unambiguous the arbitrator must accept it as final’.^59^ ‘Medics’, whether medical referees or company doctors, were thus presented as objective experts.

Medical referees, however, certainly did not always agree on the nature or extent of injuries or on the level of disablement of individuals and their ability to work. In one case where a medical assessor had advised a miner to have surgery on a severely injured hand and the miner had refused the company had subsequently withheld compensation.^60^ Another doctor had advised that the risks involved in such as operation outweighed the potential benefits. The Sheriff ruled therefore that the miner's refusal to consent to the operation was ‘reasonable’ as ‘his refusal has not proceeded from any prejudice or obstinacy of his own, but has been prompted by the skilled advice which he properly took with an open mind’.^61^ In another case a miner who had ‘racked his back’ was accused by one medical referee of exaggerating his symptoms and was later certified as unfit for any work by another.^62^

Miners were thus able, in some cases, to challenge decisions by medical referees by providing their own medical evidence. For example one miner was able to disprove the allegation that his ‘was not a genuine case’ and prove that bad ventilation in the pit had poisoned him resulting in his never being ‘fit for manual labour again’.^63^ Another miner was also able to disprove a medical referee's report when he was re-examined and certified as suffering from beat knee and as being ‘unfit for work of any kind’.^64^ In a similar way miners suffering from miner's nystagmus and beat hand were able to take their cases to Sheriff Courts and challenge medical certificates, to prove they were disabled and not fit for work and in some cases were able to get their compensation re-instated.^65^ Miner's trade unions often provided support in these cases through agents or in providing medical or legal advice. For example in the case of a forty-seven year old miner, who had suffered a crushing injury, the union agents acting for him prevented him being sent back to work underground arguing successfully that he was not fit for such work.^66^ In most cases, however, miners had to undergo a number of examinations and appeals before they were able to successfully challenge the initial decisions. They often faced a delay in compensation payments and continually had their morality and character questioned. In addition insurance companies were often involved in compensation cases. Bartrip and Burman have commented: ‘it became the practice of some companies to harass claimants in various ways, particularly into accepting large lump sum settlements’.^67^ Commuting claims into a one-off lump sum payment was a common tactic of coalowners and their mutual insurance companies (frequently at the six month point of any compensation payouts). This option could be attractive to injured and diseased miners who frequently accumulated medical expenses and other debts that needed to be paid off.

## Disability, re-employment and ‘light work’.

One common tactic deployed by mineowners and insurers to reduce their liabilities was to declare injured and disabled miners ‘partially capacitated’ or being ‘fit for light work’. Medical professionals were deployed to monitor recovery and get disabled and injured men off the compensation pay roll as soon as possible. Miners and their trade unions in turn fought against such labelling. In such cases full weekly compensation payouts could typically be halved. In a number of cases miners appealed to the Sheriff Court when they disagreed with medical referees and claimed that they were still ‘incapacitated’^68^ or ‘unfit for work of any kind’.^69^ The categorisation of ‘total’ and ‘partial incapacity’ and how this was to be judged was never particularly well defined in the original WCAs of 1897 and 1906. The findings in one case which went to the House of Lords in 1928 suggested that compensation should be paid to injured workers based on their ‘potential’ earnings as opposed to ‘actual’ earnings.^70^ There was continued contention therefore over responsibility to provide alternative ‘light work’ and the impact this should have on compensation payments. Welfarist companies such as the Fife Coal Company appear to have been more consistent than others in providing work to the disabled, including the blind. However, this was always ad hoc and discretionary (rather than a right) and became more difficult in periods of recession, such as the 1920s and 1930s.^71^ Unsuccessful bills were introduced in Parliament in 1928 and 1929 in recognition of the challenges faced by men classified as fit for ‘light work’ but unable to secure such employment noting how ‘the decline in the industry … had inflicted serious hardship upon disabled men for whom light work could not be found’.^72^

One case from Airdrie Sheriff Court was reported in the *Motherwell Times* in 1921 whereby a miner who had been claiming full compensation for injuries to his arm and side had been moved on to partial compensation and declared fit for light work.^73^ Murphy, the miner in question, reported himself unfit for this light work (carrying wood and at the picking tables—where coal was sorted and washed at the pit surface) suggesting that his injuries were an ‘odd lot’ and that he was unable to find employment in the labour market. Murphy had engaged in a number of roles that were either low paid or unsuitable and during this period he was eventually declared fit for work as a miner (though not a drawer—filling and pushing the coal tubs or wagons). Summerlee Iron Company, his employers, ultimately declared that they were ‘prepared to find him work as a miner on the face’ in order to enable them to cease compensation payments.^74^ The colliery owners seem to have been persuaded that it was in their interests to find him a suitable role to absolve them of responsibility for paying compensation. Many other miners found this categorisation of partial incapacity difficult to contend with. One miner had been on full compensation after spending two months in hospital for injuries to his wrist and ankle. However his rate was cut after the insurer's doctor declared him fit for light work.^75^ He resisted this diagnosis arguing, ‘I still have to use a stick as my foot cannot bend. And my wrist is also decidedly weak’. He also stated that his fear was that his ‘earning capacity will be reduced in the future’.^76^

The responsibility for the provision of ‘light work’ for injured or disabled men however was continually a contested area. Finding such work could be a challenge, particularly in the interwar Depression. In the case of miner Robert Laird, in 1928 it was suggested that there was still work for disabled men as although ‘the labour market had been greatly restricted for all … it has not been closed to men who are partially incapacitated’.^77^ Furthermore the Sheriff in this case stated that companies preferred to employ capable disabled men instead of continuing to pay compensation.^78^ However, the evidence in this case suggests that, in practice, securing suitable work was extremely difficult. Laird stated that ‘he had endeavoured to obtain such work but [was] mainly or wholly on account of his present condition … refused work’.^79^ An insurance agent, himself disabled through work in the mine, was also called to give evidence in this case. In response to the question of the availability of work for disabled miners he stated:

I have heard it remarked that [in some pits] they could do with men with four hands, never mind any disablement, so that a man who has a disablement is always at a disadvantage … if a man on partial compensation is asked to find light work and he goes to pits other than the pit where the accident happened, that manager would be very wary of giving him a job.^80^

Other witnesses such as the colliery manager and labour bureau manager were also called to give evidence. Whilst both argued that the company sought to find suitable work for all men regardless of their disabilities, they admitted that the lack of vacancies in the industry in general could impact heavily on the chances of disabled men seeking work and that they were often limited to applying for very specific types of jobs.^81^ An example of this from an earlier case saw an injured miner certified as ‘fit for light work which does not entail prolonged standing’.^82^ The miner, with the support of the Linlithgow Miners' Union, argued that this left him with ‘no earning capacity in the open market [given] … the limitations indicated in the Referees report’.^83^ Leslie, the manager in Laird's case, stated there were still jobs suitable for disabled men citing an example of a disabled man he currently employed who was able to work with one arm. However, he also stressed that ‘a one handed man could have little chance of competing’ when it came to applying for work.^84^ This led the Sheriff to suggest that ‘a one-handed man in any class of work for which he was fit would be handicapped in comparison with a two handed man’.^85^ In part this was linked to employers' fears that such ‘weakened’ workers were a risk, would be more liable to future accidents or proneness to disease which would add to employers’ compensation liabilities. In the interwar Depression the disabled were thus further marginalised by overstocked labour markets and the logic of workmen's compensation policies. William Steele, for example, lost his leg in an accident at Candlerigg Colliery in 1923 and was re-employed at the pit for ten years thereafter as a signaller on the pithead. After the pit closed for several weeks in 1933 he was not re-employed on reopening. The resulting Sheriff Court case and appeal upheld his claim for a full compensation award, ‘since he was fit only for a special and limited class of work which he would have no chance of obtaining in the competitive labour market’.^86^

There were reports of men facing an ‘inquisition’ upon applying for jobs in the Lanarkshire coalfields. One article in 1925 reported the difficulties men were having in finding work and a series of questions they could be asked upon application including questions on their health relating to ‘rheumatism, bronchitis or asthma’ and if they had claimed compensation in the past.^87^ The WCA had evidently brought the body under sharper scrutiny, deepening the extent of medical surveillance in the workplace. There was a reluctance to employ those with a record of workmen's compensation, such as nystagmus cases, whilst the synergistic association of silicosis with tuberculosis in the 1930s also led to dismissals fuelled by the fear of infection.^88^ This led some miners to try to hide their emerging disabilities as far as they could. One miner, who lost his appeal in the House of Lords in 1930, for example, was found guilty of making ‘false declarations in writing that he had not previously suffered from miners' nystagmus'.^89^ This miner may not have been able to find work had he declared his medical history given that ‘doctors were agreed that certain miners had a constitutional susceptibility to nystagmus while others were immune to it.’^90^

The compensation rate paid could often be reduced or compensation stopped completely if miners were able to find ‘light work’ for example at the picking tables at the surface leaving injured and disabled miners much worse off financially than before the accident.^91^ In evidence given to the Interdepartmental Committee on the Rehabilitation of Persons Injured by Accidents in 1937 a Glasgow surgeon commented on the issues relating to injured workmen and recovery for work. He noted that:

Many of these cases will naturally come before a Medical Referee, who may certify that the man is partially recovered, and that he is fit for light work, or certain forms of light work, but there is nothing to carry him further on than that. If he cannot get light work then he is stuck. We have recommended that something might be done by the statutory authority … financed by the Insurance Companies and the employers … to improve the chances of the man's final rehabilitation.^92^

He therefore suggested that this process would work by ‘reconditioning’ the men putting them through a process of ‘toughening up’ to enable them to be ready for work. Closer medical surveillance and deeper marginalisation of the disabled were features of the interwar Depression.

Mineowners and their insurers also contested Workmen's Compensation claims to repudiate liability and cut their costs. In Scotland, mineowners tactics ranged from denying the accident entirely, exploiting failure to report the accident on time, claiming pre-existing ‘exacerbating’ health conditions, alleging employee negligence (‘serious and wilful misconduct’), delaying out-payments, encouraging lump sum one-off payments and challenging the courts definition of an ‘accident’. These tactics evolved as a response to what employers regarded as encroaching state interference, with the WCA widening to include more longer term chronic conditions (such as silicosis), and rheumatism and ‘dropped foot’ on the grounds these were an ‘unintended and unexpected occurrence which produces hurt or loss'.^93^ Miners injured in the pit could have their compensation withheld or removed if they were found to have broken rules concerning safety, such as illegally riding hutches or smoking underground (‘contraband’), or had been ‘larking around’.^94^ Records of compensation negotiations offer numerous examples of companies trying to reduce compensation pay-outs, even in serious cases where they admitted liability and were in agreement about the severity of the injuries.^95^ Robert Allan, for example, lost his leg in an accident with a hutch on the pithead during the First World War. The medical referee stated that Allan would never be fit to wear an artificial limb and would most likely die within four or five years. Nonetheless, the company held out with an offer of £100 lump sum compensation—half of what Allan had expected and stated he would accept.^96^ This adds weight to the argument that there was a distinctive and different story in the Scottish coalfields, where social relations were markedly more conflictual. In this respect Scotland had more in common with South Wales. In contrast to the North East of England, it is significant that formal arbitration over disputed medical assessments and compensation payments did not exist in Scotland.^97^ This co-operation in North East England had its roots in traditions of joint administration within Permanent Provident Funds between masters and men and formal Arbitration Committees which prevented many of these cases going to court.^98^

## Challenging dominant discourses: Advocacy for disabled men

Miners and their trade unions in Scotland played an important part in shaping the discourses and struggles over workmen's compensation. Bartrip has argued that many workers were ignorant or ‘misinformed’ of compensation legislation, especially where trade unionism was weak.^99^ Thus, it could happen that accident wounds went septic before being reported.^100^ In some cases, letters were written to newspapers for advice on compensation and levels of entitlement.^101^ One such case in 1932 was from a miner with amputated fingers who stated ‘I will never again be able for my old work’. He was advised of the levels of weekly payments he could be entitled to and the lump sum payment he should seek.^102^ Another wrote in concerning his ‘beat knee’ stating that his panel doctor had advised him to report to the certifying doctor as soon as possible in order to claim compensation.^103^ To some extent then, miners were taking responsibility for educating themselves about their entitlements, drawing upon wider sources of support than their trade unions.

Nonetheless, the coal miners were one of the most well organised of all occupational groups, demonstrated not just in very high union densities (the proportion of the total workforce that were unionised), but also in high levels of strike activity. Trade union membership was over 50% for coal miners in the UK in the late nineteenth century, rising to around 90% by Second World War.^104^ Scottish miners were amongst the most unionised and amongst the most militant. Trade union density was calculated by Campbell to be over 70% in Scottish coalfields such as the Lothians and Ayrshire by 1910.^105^ Bartrip's point about workers' being ignorant about aspects of workmen's compensation where unions were weak certainly did not apply in this context.^106^ That said, there was a culture of manliness and socialisation or acculturation to high levels of risk in underground mining that could undermine health over the long term and which sometimes contributed to injuries not being reported and compensation claims not being initiated.^107^ The evidence nonetheless strongly points to a very proactive trade union strategy on compensation *and* prevention in coal mining that challenged medical orthodoxies and managerial compensation and malingering discourses.

The Scottish mining unions appear to have developed an extremely dynamic advocacy role on behalf of disabled mineworkers, campaigning actively and aggressively to protect and advance their interests. They developed a range of arguments to challenge claims of shirking and dynamic tactics to mitigate the poverty and social exclusion that invariably went along with disability through representing ‘victims’ in compensation cases that provided some (albeit limited) wage substitute in the case of permanent disability, and raised the likelihood of re-employment. For miners this facilitated the maintenance of self-esteem, independence and gender norms and identities associated with working-class ‘provider’ masculinity. There were a number of layers to this trade union discursive and material activity: raising awareness and levels of knowledge; advocacy and material support in compensation cases and campaigning; lobbying and policy-making. The unions disseminated information about workers' rights under the WCA, encouraged the reporting of injuries and disease and supported cases where employers or their insurance companies contested liability. There are numerous reports of trade unions taking on particular cases where miners were refused compensation to advocate on their behalf. The Scottish Miners' Federation also campaigned for changes to the compensation legislation for disabled miners who were often at the whim of employers and their insurance companies over lump sum payments, or doctors over recommendations on length of recovery. Scottish mining trade unions also promoted the case for higher payments and fairer compensation pay outs. The Scottish Union of Mineworkers for example highlighted the impact that rampant wartime price inflation had upon compensation levels and sent a recommendation to the Coal Commission in 1919 to increase payments to disabled men by 100% to address this.^108^

The voluminous coal mining trade union records point to a high level of awareness and advocacy on behalf of disabled miners in this period at two levels: the workplace community and in policy-making. In 1920, for example, the Miners' Federation of Great Britain (MFGB) reflected on the difficulties and ‘hardship’ of two categories of disabled people in mining communities: the ‘light work compensation men’; and the ‘permanently disabled workmen’.^109^ The latter, the Chairman of the MFGB Executive Committee noted, received a ‘totally inadequate amount of compensation’. Commonly, benefit levels were set at around £1 per week (around half of basic wages) when actual earnings of face workers were around £3 and £4 per week. At the local level injured miners were supported in making claims and contesting cases through the Sheriff Courts and, with the support of the MFGB, appeal cases to the House of Lords. In such cases the miners' unions would pay for legal counsel and base a judgement on pursuing a case, or making an appeal on this ‘expert’ opinion. In two Scottish cases in 1919–20, for example, the MFGB agreed to support cases of serious disablement of shotfirers (loss of sight in one case and loss of a leg in another) despite ‘contributory negligence’ or breach of safety rules being alleged. In at least one of these cases partial compensation (of 17*s*. 6*d*.) was awarded by Sheriff John Guy despite ‘serious and wilful misconduct’.^110^ The shotfirer in question was said to have prior earnings of around £4 per week.

Trade union appeals on behalf of this growing and more visible disabled community were frequently framed within the language of a moral economy with reference to ‘social justice’ and common humanity. The President of the MFGB, the Scot Robert Smillie, commented in November 1918:

I do not know any people who have been worse hit by the national crisis through which we are at present passing than those that have the misfortune to be wholly disabled. In many of the houses they have been practically starving. Local assistance has been given by the miners' organisations or by charitable persons.^111^

The Scottish mining unions campaigned (unsuccessfully) for the state provision of artificial limbs and statutory rights to alternative employment—‘the kind of light work a workman is certified fit for’.^112^ The more radical voices in the industry called for direct strike action ‘to raise the bottom dog in our industry, our compensation men’.^113^ However, this appeal to down tools was never actually taken up amidst accusations that the MFGB put the interests of the able-bodied before the disabled. Whilst there were individual seam and pit-level walkouts on issues like dust, occupational health and safety strikes were a rare occurrence even in the more militant British coalfields.^114^

The evidence then indicates that the mining unions were playing a significant role in shifting the discursive terrain towards ideas of social justice and state responsibility for occupational risk and workers' welfare. They were campaigning for preventative measures (such as the Mining Acts) and mediating between the disabled worker and the market, whilst mitigating, to some extent, the impact of disabling injuries and chronic disease. The compensation base income was raised by 25% in 1917 as a response to inflation—though on the grudging condition that the issue was not to be returned to for the duration of the war. When the MFGB did go back for more in October 1918 the mineowners rejected this outright, though the state intervened with the War Additions Act raising workmen's compensation benefits by some 75% for disabled workers.^115^ Still, it was estimated that comparing 1914 with 1920, disabled miners in receipt of workmen's compensation benefits were around 30% worse off due to price inflation.^116^

The interwar years of contraction in coal mining, characterised by pit closures, mass unemployment and short-time working were an extremely difficult period for the disabled in Scottish mining communities. Scotland experienced significantly higher levels of poverty than England between the wars and the wider context was one of growing deprivation and dependency upon the state. As Levitt has shown, numbers on Poor Relief under the Poor Law in Scotland tripled between 1920 and 1938.^117^ Economic circumstances underlay a marked shift in power from the miners and their unions to the coalowners and the employers' movement. The scene was set for a wide-ranging employers' offensive against organised labour which neutered ‘Red Clydeside’. One element of this was the removal of the most active, radical elements from workplaces in a victimisation and blacklisting campaign the ferocity of which to some extent was disguised by sharply rising unemployment levels. Some Scottish union activists—for example the McGahey family—had to move as far as the Kent coalfield (with its notoriously wet working conditions) to find work. The bargaining power of the miners' unions eroded sharply in the early 1920s, culminating in the defeat of the General Strike and the miners' lock-out that followed. Anti-union legislation in 1927 further undermined the capacity of the unions to protect members' interests in the workplace and the community.

The union records suggest activities on behalf of the disabled in mining communities continued at a significant level, but that union operations proceeded under severe constraints in a difficult environment during the Depression. Defensive strategies designed to protect working miners' jobs and wage levels appear to have been prioritised. In a period before the welfare state when jobs and the income they provided were fundamental to maintaining health and well-being this was pragmatic and understandable. Still, it appears, injured and diseased members continued to be supported in their claims for compensation, with the unions contesting and challenging employers and insurance companies attempts to deny liability and minimise payments. Scottish cases continued to be financially supported through the law courts and up to the House of Lords if a precedent was considered important to sustain.^118^ But the momentum had shifted with the transition in power that came with mass unemployment. As early as 1921 James Murdoch of the National Union of Scottish Mineworkers alerted the Scottish TUC to a tougher line amongst Scottish collieries compared to the English on the re-employment of disabled miners on ‘light work’.^119^ It was noted that the mine owners were initiating more and more compensation appeals to cut their costs and that the proportion that the unions could challenge was diminished because of the dire position of their finances after the 1926 General Strike and lock-out and with the membership haemorrhage consequent upon mass unemployment.^120^ Moreover, union appeals for a wholesale reform of workmen's compensation fell on deaf ears until the welfare reforms of the 1940s. The coal owners meanwhile threatened to end the Miners' Welfare Fund (which was supported from a levy on coal tonnage)—which amongst other things was used to provide specialist medical and rehabilitation treatment in serious accident cases. Mineowners also exploited a conservative medical boards and appeals system to declare disabled miners ‘capable of light work’, thus cutting benefits.^121^ As already noted, those shifted from full to ‘partial’ disability in this way struggled to find work as vacancies evaporated. The MFGB took an appeal to the House of Lords to argue that as no work was available then this process was effectively denying the right to compensation—but this was rejected.^122^ Fortunately for disabled miners, most collieries were insured against workmen's compensation risks. In some cases, however, where companies went bust compensation payments ceased when assets were exhausted.^123^ Because compensation rates were based on previous actual earnings, the amounts disabled miners had to live on were also eroded by several wage cuts imposed upon miners in the Depression from 1921, by short-time working and by the application of the Means Test to benefits after 1931.^124^ Growing levels of non-unionism left some miners particularly vulnerable. The Lanarkshire Miners' Union identified the proliferation of ‘touts’ who were reported to have been ‘pestering the relatives of injured miners … persuading them to enter into actions involving heavy expenses’.^125^ The ‘touts’ were reported to prey on non-unionised, presumably ill-informed and more vulnerable miners and to pocket much of the subsequent compensation payments. Where miners worked directly for smaller contractors there are also examples of miners failing to claim for injuries and of compensation being stolen.^126^

To address new challenges in the interwar years, the miners' unions adapted campaigns, switching to demands for the state to take over full liability for the workmen's compensation system and the ‘internationalising’ of workmen's compensation through the Miners' International Federation Congresses and the International Labour Organisation.^127^ Significantly, Britain failed to ratify the 1925 International Convention on Workmen's Compensation before the Second World War.^128^ By the end of the 1930s Scottish miners' unions regarded the workmen's compensation system as ‘totally unsatisfactory’, lambasting it as an iniquitous scheme that lined the pockets of lawyers and doctors, whilst failing to provide for any rehabilitation or have any significant preventative impact.^129^ Testimony to the sustained advocacy role of the unions is the fact that over 20% of the resolutions taken through the annual conference of the Scottish miners in 1940 related to disability, including a demand for single medical referees to be replaced by a panel of three, compensation to be set at 75% of ‘normal pre-accident weekly earnings’, coal to be supplied at cost price to ‘sons and daughters of householders who are disabled or retired’ and the WCA widened to cover ‘all diseases arising from employment’.^130^ At a local level the Scottish miners' unions paid medical fees and legal fees associated with all compensation cases and union representatives met with the General Manager of the Mineowners' Defence and Mutual Insurance Association in Glasgow to press the claims of disabled miners.^131^ Some trade unions also provided artificial limbs to amputee miners after collecting money from other workers and at public events such as football matches.^132^

The injured body continued to be a point of contestation in the law courts in Scotland, with varying outcomes during the Depression. The Sheriffs appear not to have been the uncritical mouthpiece of local elites and were capable of interpreting legal precedents in WCA cases and making independent decisions. For example, there were a series of cases in Scotland (and elsewhere) over the compensation rights of coal face miners (hewers and machine men) who had lost one eye. The custom was for such miners to be re-employed on their recovery and compensation payments stopped on the grounds they were ‘as good as new’. This was challenged in the courts. At the final stage of appeal in the case of Burt vs Fife Coal Company Lords Johnston and Skerrington found in favour of the workman on the grounds of greater risk, ‘owing to the workman only having one eye the consequences of an accident to that eye would be very much more serious than if he had two eyes'.^133^ Where disabled workers were re-employed and subsequently lost work, courts upheld appeals for a return to full levels of compensation—as in the case of Walker vs Wemyss Coal Company in 1929 and a case heard in the Hamilton Sheriff Court in 1935.^134^ Elsewhere, however, a harder line was evident. In a Stirling Sheriff Court case in 1934 personal factors—including ‘bad teeth’ and ‘want of exercise’—were deemed to have affected a workplace back injury and compensation reduced, supporting the employers' case.^135^ Nystagmus cases were also appealed by coal owners who claimed that the effects on vision were not such that prevented them from continuing to work at the coal face and maintaining full ‘earning capacity’—as in a Linlithgow Sheriff Court case in 1930.^136^

One important change that the interwar years witnessed was the redefining of respiratory disability. There were significant (if limited) successes in getting the causal pathways of dust-related miners' diseases identified. ‘Miners’ asthma’ and ‘black lung’ had been widespread within many mining communities through the nineteenth century and the wheezing and coughing miner, struggling for breath and having difficulty walking was commonplace. The first official recognition of silicosis came in 1918 when it was added to the list of prescribed occupational diseases under the WCA. Miners, however, were initially excluded. Even after 1928 when the legislation was amended to include miners, in practice there remained severe restrictions upon miners making any claims, including the requirement to prove at least 50% silica content in the rock being worked underground. The difficulty lay in the fixation within the medical community upon silica as the only damaging agent in respiratory impairment. Carbon (coal) dust was considered innocuous; even in some quarters a prophylactic. Progressively silicosis and coal workers' pneumoconiosis (CWP) were defined as occupation-related disabilities in a long campaign by the miners' unions and sympathetic medical supporters. But CWP (or ‘black lung’) was not made compensatable until 1943 and the long delay from identification in the medical literature (albeit contested) of coal dust as an agent and the addition of CWP to the list of prescribed industrial diseases under the WCA resulted in enormous hardship for those coal miners with respiratory disabilities before the Second World War who could not claim workmen's compensation. In South Wales this has been described as ‘devastating’ and ‘a nightmare for the disabled pneumoconiotic miner’.^137^ This story has been reconstructed in some detail in recent research.^138^ A key point that emerges from these studies is the resilience of the miners' unions and their key role at the workplace, community and policy-making levels in advocating for the disabled and campaigning to reform workmen's compensation to include silicosis and, belatedly, CWP in 1943. Because of the feared (and much misunderstood) association between silicosis and tuberculosis (which was dynamically synergistic) and uncertainty over the infective nature of the disease, known silicotics were invariably sacked in the 1920s and 1930s. Alternative job opportunities in mining communities were difficult for the able-bodied in the Depression, never mind the disabled, as the Medical Inspector of Mines, S. W. Fisher, pointed out in 1931.^139^ Those miners diagnosed with silicosis and with severe respiratory disabilities mostly joined the TB ‘lungers’— excluded from work, emasculated and stigmatised. Others hid their disability, did not claim compensation and continued working for as long as possible, inhaling the toxic dust and in the process exacerbating their progressive fibrosis of the lungs further.

## Conclusion

Working in coal mining was a risky endeavour before the Second World War which mangled up bodies, damaging beyond repair miners eyes, joints, limbs, muscles and backs, whilst the labour process and polluted work environment was directly responsible for a series of health-undermining chronic industrial diseases. The workmen's compensation scheme was established in 1897 to address the social consequences of this carnage in the workplace. Moses has advocated for research on occupational risk and workmen's compensation to drill down to the national and local level^140^ and this article has explored the discourses around disability that circulated through the heyday of the workmen's compensation system in the interwar period and the contestation that characterised this element of the welfare state in the Scottish workplace. The evidence reveals how ‘new’ and long-standing disabilities (such as repetitive musculo-skeletal disorders and respiratory disease) were redefined as occupational and how the system strengthened the social model of disability, associating, as it did, liability and responsibility with industry and disability with restricted functionality relating to work tasks and work environments. In this context, disability was not a fixed construct but a highly contested one where stakeholders framed narratives of disability to support their arguments for or against financial compensation awards. A prevailing narrative was that those disabled by workplace injuries or disease had to adapt to their changed circumstances; there was little or no onus on mechanical adaptations and reorganisations of labour processes in this period.

Workmen's compensation was symbolically important and marked a step change in the rights of disabled workers, providing a clear line of responsibility back to industry and undoubtedly ameliorating the economic circumstances of injured and disabled individuals and their families. It appears to have placed a significant financial inducement upon coal owners to provide alternative employment to categories of disabled miners. Its operation in practice, however, explored through the prism of Scottish mining communities, highlights a contested, adversarial system which brought workers, employers and their representatives into conflict, drawing medicine into making judgements about the extent and significance of disablement and lining the pockets of insurance companies and lawyers. This led to the morality of the disabled being questioned in discourses over ‘malingering’. Whilst medical opinion ranged widely across a broad spectrum, medicine was co-opted by both sides to support arguments, raising questions over the ‘neutrality’ of medical expertise in this context.

A key point that emerges from this analysis is the extent to which disability became more visible, more closely monitored and a site of contestation and struggle over compensation within Scottish mining districts between the wars. How this worked through at the local level, in medical examinations, within trade unions and in the Sheriff Courts in Scotland, reveals much about the diverging and competing definitions of disability within industry, medicine and the state. These sources also provide glimpses of the meaning of disability in these contexts and of lived experience; of the material struggles and hardships of the men and their families directly affected. The system brought closer medical surveillance to mining communities, with bodies under more intrusive scrutiny, as well as increased sensitivity by the mine owners to the employment of diseased, disabled and older, more vulnerable workers who might be a compensation risk. Whilst Workmen's Compensation provided a welcome financial buffer for some, for others it represented the reason why they were deprived of employment and denied the economic rewards and social inclusion work provided. In response, some opted to hide their disabilities from the medical-managerial gaze and continue working for as long as possible.

This investigation of workmen's compensation in the Scottish coalfields has also highlighted the role of the disabled and their advocates in shaping the system and challenging the discourses of the coal owners, medicine and the state.^141^ What emerges from the evidence is the key role the mining trade unions played as dynamic advocates for disabled miners, disseminating information, advising and educating, supporting claims, representing the disabled, financing medical examinations and paying legal fees. In the process, the unions were accumulating an alternative body of knowledge, challenging orthodox medical opinion and discourse and developing a network of supporters in local government, medicine and politics. They were learning how to play the compensation game, bringing bargaining skills accumulated in adversarial industrial relations and collective bargaining over wages and conditions into the medical arena. There were many layers to the supportive matrix for the disabled provided by the miners' trade unions, from pit level and local advocacy and campaigns, to national movements to widen definitions of occupational disability and fundamentally reform workmen's compensation. That said, trade union advocacy for the disabled was contingent, influenced and constrained by a whole raft of factors and circumstances, including shifting power dynamics that ebbed and flowed with prevailing labour market and other circumstances. In Scotland, the Depression appears to have deepened the adversarial and confrontational nature of struggles over definitions of disability and compensation. The Scottish evidence, moreover, demonstrates the agency of miners themselves in disability politics and suggests, to us at least, that more attention needs to be paid to interrogating the role of the trade unions in the history of disability and health than has hitherto been the case.

## Notes

2 Henry Louis, ‘Mining’ in Thomas Oliver (ed.), *The Dangerous Trades* (London, 1902), 530–2; Roy Church, *The History of the British Coal Industry, vol 3* (Oxford, 1986), 303, 306.

3 Parliamentary Papers [PP], 1925, XVI (Cmd. 2600), *Report of the Royal Commission on the Coal Industry*, 191–2.

4 PP, 1938 (Cmd 5890), *Report of the Royal Commission on Safety in Coal Mines*, 60.

5 P. W. J. Bartrip and S. B. Burman, *The Wounded Soldiers of Industry: Industrial compensation policy*, *1833–1897* (Oxford, 1983); P. W. J. Bartrip, *Workmen's Compensation in Twentieth Century Britain: Law, history and social policy* (Aldershot, 1987); Joseph Melling and Mark Bufton, ‘“A mere matter of rock”: Organised labour, scientific evidence and British government schemes for compensation of silicosis and pneumoconiosis among coalminers, 1926–1940’, *Medical History* 49 (2005) 155–78; Julia Moses, ‘Foreign workers and the emergence of minimum international standards for the compensation of workplace accidents, 1880–1914’, *Journal of Modern European History* 7 (2009) 219–239.

6 Arthur McIvor and Ronald Johnston, *Miners*' *Lung: A history of dust disease in British coal mining* (Aldershot, 2007); Mark Bufton and Joseph Melling, ‘“Coming up for air”: Experts, employers and workers in campaigns to compensate silicosis sufferers in Britain, 1918–1939’, *Social History of Medicine* 18 (2005) 63–86. See also Michael Bloor, ‘No longer dying for a living: Collective responses to injury risks in South Wales mining communities, 1900–1947’, *Sociology* 36 (2002) 89–105.

7 The phrase ‘injured workers’ movement’ has been used in the context of Canada. See Robert Storey, ‘Beyond the body count? Injured workers in the aftermath of deindustrialization’, in Steven High, Lachlan MacKinnon and Andrew Perchard (eds), *The Deindustrialized World* (Vancouver, 2017), 46–67.

8 See, for example, Ian Levitt, *Poverty and Welfare in Scotland, 1890–1948* (Edinburgh, 1988); Jacqueline Jenkinson, *Scotland's Health, 1919–1948* (Oxford, 2002).

9 Alan Campbell, *The Scottish Miners, 1874–1939*, 2 vols (Aldershot, 2000).

10 Leah Leneman, ‘Wemyss Coal Company 1906–24’, *Scottish Economic and Social History* 13 (1993) 54.

11 Anne Borsay, *Disability and Social Policy since 1750* (New York, 2005), 10–14.

12 Brendan Gleeson, *Geographies of Disability* (London, 1999), 106.

13 See Mike Mantin, ‘Coalmining and the National Scheme for Disabled Ex-Servicemen after the First World War’, *Social History* 41 (2016) 155–170.

14 Julie Anderson, *War, Disability and Rehabilitation in Britain, ‘Soul of a Nation’*, (Manchester, 2011), 24; See also Julie Anderson, ‘Marks on men’, Unpublished public lecture, University of Strathclyde, 21 May 2014.

15 Joanna Bourke, *Dismembering the Male: Men's bodies, Britain and the Great War* (London, 1996), 37; Noelle Whiteside, ‘Counting the cost: Sickness and disability among working people in an era of industrial recession, 1920–39’, *Economic History Review* 40 (1987) 241.

16 Julia Moses, ‘Contesting risk: Specialist knowledge and workplace accidents in Britain, Germany and Italy, 1870–1920’, in Kerstin Brückweh, Dirk Schumann, Richard Wetzell and Benjamin Ziemann (eds), *Engineering Society: The scientification of the social in comparative perspective, 1880–1990* (Basingstoke, 2012), 219–37; Moses, ‘Foreign workers’, 219–239. See also Robert Asher, ‘Experience Counts: British workers, accident prevention and compensation, and the origins of the welfare state’, *Journal of Policy History* 15 (2003) 359–388; Catherine Mills, *Regulating Health and Safety in the British Mining Industries, 1800–1914* (Farnham, 2010).

17 Vicky Long, *The Rise and Fall of the Healthy Factory; The politics of industrial health in Britain, 1914–1960* (Basingstoke, 2011); Bloor, ‘No longer dying’; Melling and Bufton, ‘A mere matter of rock’; McIvor and Johnston, *Miners*' *Lung*.

18 Miners' Federation of Great Britain [MFGB], *Annual Conference Proceedings*, 9 Aug. 1918, 87.

19 *Ibid*., 93.

20 See Campbell, *Scottish Miners*, i. 238; McIvor and Johnston, *Miners*' *Lung*, 259–69.

21 For a discussion of what constituted a workplace accident see Karl Figlio, ‘What is an accident?’ in Paul Weindling (ed.), *The Social History of Occupational Health* (London, 1985), 180–206.

22 Moses, ‘Contesting risk’, 60.

23 See Arthur McIvor, *Organised Capital* (Cambridge, 1996), 79–81. For a discussion of prevention and compensation politics in an earlier period in mining see Mills, *Regulating Health*.

24 However, silicosis was initially (1918) very narrowly defined under the WCA and coal miners almost entirely exempt. Coal workers' pneumoconisis was not officially recognised and added to the list of statutory diseases under the WCA until 1943.

25 This was later reduced to seven days and down to four days in 1923.

26 Edinburgh, National Records of Scotland [NRS], CB19/2/F608/15: Patrick McLaughlin vs The Fife Coal Co Ltd, Scottish Coal Workers' Compensation Scheme, Directors Minute Book 3, Dunfermline Office, 1914–16.

27 NRS, SC21/12A/1928/18: Robert Laird vs The Fife Coal Company, Dunfermline Sheriff Court, 18 Sep. 1928 and 8 Nov. 1928.

28 *Ibid*.

29 NRS, CB19/2/ F2774/16: Archibald Arnott vs The Fife Coal Co Ltd, Scottish Coal Workers' Compensation Scheme, Directors Minute Book 3, Dunfermline Office, 1914–16. A ‘machineman’ was an underground coal face worker directly responsible for working a power-driven mechanical coal-cutting machine which ‘under-cut’ the coal seam.

30 NRS, CB19/6/ F4460/34: Millar vs Wemyss Coal Co Ltd, Scottish Coal Workers' Compensation Scheme, Directors Minute Book 10, Dunfermline Office, 1933.

31 NRS, SC67/78/1926/17: Robert Low vs Plean Colliery Company Ltd, Stirling Sheriff Court, 27 Mar. 1926.

32 Bourke, *Dismembering the Male*, 66–7.

33 Anderson, *War, Disability and Rehabilitation*, 5.

34 Anne Borsay, ‘Disciplining disabled bodies: The development of orthopaedic medicine in Britain, *c*.1800–1939’, in David Turner and Kevin Staggs (eds), *Social Histories of Disability and Deformity* (Abingdon, 2006), 105.

35 Anderson, *War, Disability and Rehabilitation*, 7.

36 NRS, CB19/2/F1959/15: John Simpson vs The Wemyss Coal Co Ltd, Scottish Coal Workers' Compensation Scheme, Directors Minute Book 3, Dunfermline Office, 1914–16. A brusher was employed to cut or blast rock or coal from a roof or face.

37 Moses, ‘Contesting risk’, 69–70.

38 Bourke, *Dismembering the Male*, 88–9.

39 As asserted by the medical representative of the West Scotland Engineering Employers' Association to the Departmental Committee on Compensation for Industrial Diseases, Minutes of Evidence, PP, 1907, (Cd 3496), Q5166–68, 163.

40 Bourke, *Dismembering the Male*, 102.

41 Bartrip, *Workmen's Compensation in Twentieth Century Britain*, 70–1.

42 Alexander Renfrew, ‘Mechanisation and the Miner: Work, Safety and the Labour Relations in the Scottish Coal Industry, 1900–1939’, unpublished PhD thesis (University of Strathclyde, 1997).

43 Bartrip, *Workmen's Compensation in Twentieth-Century Britain*, 134. There were over 6,000 contested cases taken to court every year from 1922–31.

44 Leneman, ‘Wemyss Coal Company’, 49. See also Bartrip, *Workmen's Compensation in Twentieth-Century Britain*, 54.

45 Leneman, ‘Wemyss Coal Company’, 51.

46 *Times*, 19 Oct. 1910.

47 Archibald McKendrick, *Malingering and its Detection under the Workmen's Compensation and Other Acts* (Edinburgh, 1912), Preface, 7.

48 *Ibid*., 68.

49 NRS, CB19/2/ F.2370/13: James Hunter vs The Fife Coal Co, Scottish Coal Workers' Compensation Scheme, Directors Minute Book 3, Dunfermline Office, 1914–16.

50 *Ibid*.

51 William Fletcher, *The Mining Association of Great Britain, Workmen (Compensation for Accidents) Bill* (London, 1897), 8.

52 *Ibid*., 8.

53 ‘Motherwell miner's effrontery: Thirty days for fraud’, *Motherwell Times*, 12 Dec. 1924.

54 ‘Miner's fraud’, *Evening Telegraph* (Dundee), 15 Sep. 1926.

55 Bourke, *Dismembering the Male*, 48.

56 Bartrip, *Workmen's Compensation in Twentieth-Century Britain*, 74.

57 McKendrick, *Malingering*, 78.

58 See Melling and Bufton, ‘“A mere matter of rock”, 155–78; McIvor and Johnston, *Miners Lung*, 71–5, 187–9.

59 ‘Medico-legal: Workmen's compensation: Effect of medical referee's certificate’, *British Medical Journal* [BMJ], 3 Aug. 1929, 228.

60 ‘Doctors differ as to operation’, *The Post*, 4 Mar. 1917.

61 *Ibid*.

62 NRS, CB19/4/ F426/20: Scottish Coal Workers' Compensation Scheme, Directors Minute Book 6 , Dunfermline Office, 1919–21.

63 NRS, CB19/2/F.3897/16: James Sherry vs The Fife Coal Co Ltd, Scottish Coal Workers' Compensation Scheme, Directors Minute Book 3, Dunfermline Office, 1914–16.

64 NRS, CB19/6/ F237/34: Ferguson vs Wilsons and Clyde Coal Co Ltd, Scottish Coal Workers' Compensation Scheme, Directors Minute Book 10, Dunfermline Office, 1933.

65 NRS, SC67/78/1926/3: James Quinn vs Archibald Russell Limited, Sheriff Court Stirling, 30 Dec. 1925; NRS, SC67/78/1925/1: Alexander Montgomery vs Alloa Coal Company Limited, Sheriff Court Stirling, 30 Dec. 1926.

66 NRS, CB19/4/F831/18: Hetherington vs The Fife Coal Co Ltd, Scottish Coal Workers' Compensation Scheme, Directors Minute Book, Jan. 1919–May 1921.

67 Bartrip and Burman, *Wounded Soldiers of Industry*, 211.

68 NRS, CB19/2/ F630/15: Peter Donoghue vs The Fife Coal Co Ltd, Directors Minute Book 3, Dunfermline Office, 1914–16.

69 NRS, CB19/2/ F337/15: Ebenezer Sinclair vs The Lochgelly Iron and Coal Co Ltd, Directors Minute Book 3, Dunfermline Office, 1914–16.

70 Bartrip, *Workmen's Compensation in Twentieth-Century Britain*, 153–4.

71 Augustus Muir, *The Fife Coal Company Limited: A short history* (Cambridge, 1952), 80.

72 Bartrip, *Workmen's Compensation in Twentieth Century Britain*, 155.

73 ‘Bellshill miner's award’, *Motherwell Times*, 7 Mar. 1924.

74 *Ibid*.

75 ‘Compensation cases: Miner's broken wrist and ankle’, *Edinburgh Evening News*, 27 Jul. 1932.

76 *Ibid*.

77 NRS, SC21/12A/1928/18: Robert Laird vs The Fife Coal Company, Dunfermline Sheriff Court, 18 Sep. 1928 and 8 Nov. 1928.

78 *Ibid*.

79 *Ibid*.

80 *Ibid*. Evidence given by Alexander Cant, Insurance Agent.

81 *Ibid*.

82 NRS, CB19/4/ F645/19: James Caldwell vs The Fife Coal Company Ltd, Scottish Coal Workers' Compensation Scheme, Directors Minute Book 6, Dunfermline Office, 1919–21.

83 *Ibid*.

84 NAS, SC21/12A/1928/18: Robert Laird vs The Fife Coal Company.

85 *Ibid*.

86 *Colliery Guardian*, 11 Oct. 1935.

87 ‘Inquisition on miners’, *The Post*, 24 May 1925.

88 See Arthur McIvor, ‘Germs at work: Establishing tuberculosis as an *occupational* disease in Britain, *c*.1900–1951’, *Social History of Medicine* 25 (2012).

89 ‘Lords dismiss Scots miner's appeal’, *Dundee Courier*, 29 Jul. 1930.

90 ‘Hamilton miner's claim’, *Evening Telegraph* (Dundee), 30 Mar. 1932.

91 For examples see ‘Miner's compensation reduced’, *The Post*, 8 Jan 1922; ‘Miner's poisoned knee’, *Motherwell Times*, 21 Nov. 1924.

92 Glasgow, Royal Society of Physicians and Surgeons Archives [RSPSG], 39/6/8 (Young Papers): Transcript of evidence from Archibald Young (Regis Professor of Surgery, Glasgow University and Visiting Surgeon Western Infirmary) to the Interdepartmental Committee on the Rehabilitation of Persons Injured by Accidents (14^th^ Meeting, London, 30 Sep. 1937).

93 ‘Medico-legal: Dropped foot as an accident’, *BMJ*, 18 May 1940, 835; ‘Miner refused compensation’, *The Post*, 15 Jan. 1922; ‘Medico-legal: Workmen's compensation for rheumatism. Glasgow Coal Company Ltd vs Welsh’, *BMJ*, 25 Mar. 1916, 470.

94 ‘Injured while larking’, *The Post*, 15 Feb. 1920; ‘Injured riding a coal hutch’*, The Post*, 1 Mar. 1925; ‘£1 a week for a miner’, *The Post*, 18 Mar. 1917; ‘Miner who smoked’, *The Post*, 25 Jul. 1920; ‘Smoking in pit case’, *The Post*, 9 Jan. 1921; ‘Delay in paying compensation’, *The Sunday Post*, 16 Jan. 1921.

95 NRS, CB19/1–13: Scottish Coal Workers' Compensation Scheme, Directors Minute Books 1912–43.

96 NRS, CB19/2/ F.1353/15: Robert Allan vs The Earl of Rosslyn's Colliers Ltd, CB19/2/ F.2370/13: James Hunter vs The Fife Coal Co, Scottish Coal Workers' Compensation Scheme, Directors Minute Book 3, Dunfermline Office, 19–16.

97 Ian MacDougall (ed.), *Mid and East Lothian Miners*' *Association Minutes, 1894–1918* (Edinburgh, 2003), 302, 307.

98 Sunderland, Sunderland University Special Collections, NUMDA/6/1/1/3, Durham Miners Association, Report of Proceedings of Arbitration Committee Meeting held in the Coal Trade Offices, Newcastle-on-Tyne 6 Feb. 1922; and Durham, Durham Archives, D/DMA (Acc: 1004(D)) 169, Durham Miners' Association, Compensation Department monthly reports, Jan.–Dec. 1922.

99 Bartrip, *Workmen's Compensation in Twentieth-Century Britain*, 129. See also ‘Miner misinformed’, *The Post*, 11 Oct. 1942.

100 Leneman, ‘Wemyss Coal Company’, 49.

101 For example, ‘Compensation cases: Miner's broken wrist and ankle’, *Edinburgh Evening News*, 27 Jul. 1932.

102 ‘A miner's hurt hand’, *Edinburgh Evening News*, 10 Aug. 1932.

103 ‘Miner's knee injury’, *Edinburgh Evening News*, 25 Sep. 1932.

104 Arthur McIvor, *A History of Work in Britain, 1880–1950* (Basingstoke, 2001), 202.

105 Campbell, *Scottish Miners, 1874–1939*, ii. 44.

106 Bartrip, *Workmen's Compensation in Twentieth-Century Britain*, 129. See also ‘Miner misinformed’, *The Post*, 11 Oct. 1942.

107 One outcome was that minor wounds could become septic. See Leneman, ‘Wemyss Coal Company’, 49.

108 ‘Scottish miners want increased compensation: Appeal to the Coal Commission’, *Evening Telegraph*, 7 Apr. 1919.

109 Miners' Federation of Great Britain, *Annual Report* [MFGB, *AR*], 1920, 1465.

110 MFGB, *AR*, 1920, 1034–5. A shotfirer was responsible for preparing and igniting explosive charges underground.

111 MFGB, *Deputation to the Home Secretary*, 1 Nov. 1918 in MFGB, *AR*, 1918, 3.

112 MFGB, *Annual Conference Proceedings*, 9 Aug. 1918, 95; MFGB, *AR*, 1920, 702.

113 MFGB, *Annual Conference Proceedings*, 9 Aug. 1918, 704.

114 On this see Arthur McIvor, ‘Was Occupational Health and Safety a Strike Issue? Workers, unions and the body in twentieth-century Scotland’, *Journal of Irish and Scottish Studies* 8 (2017).

115 MFGB, *Deputation to the Home Secretary*, 1 Nov. 1918 in MFGB, *AR*, 3.

116 MFGB, *Annual Conference Proceedings*, 6 Jul. 1920, 99–101.

117 Levitt, *Poverty and Welfare*, 207.

118 As for example in Connor vs the Cadzow Coal Co. in 1929–30, reported in MFGB, *Annual Volume of Proceedings*, 1929–30, 1383.

119 Scottish Trade Union Congress, *Twenty-Fourth Annual Report*, 20–23 Apr. 1921, 76–7.

120 MFGB, *Annual Volume of Proceedings*, 1929–30, 110.

121 See MFGB, *Annual Volume of Proceedings*, 1929–30, 506. On the Medical Boards, see Geoff Tweedale and Philip Hansen, ‘Protecting the workers: The Medical Board and the asbestos industry, 1930s–1960s', *Medical History* 42 (1998) 439–57.

122 MFGB, *Annual Volume of Proceedings*, 1929–30, 110–11.

123 MFGB, *Annual Volume of Proceedings*, 1929–30, 659.

124 National Union of Scottish Mineworkers [NUSM], *Annual Conference Minutes*, 2–4 May 1940, 8.

125 ‘War on the touts’, *The Post*, 27 Jan. 1935. The issue resurfaced in Second World War at the Gleneagles Miners' Rehabilitation Centre.

126 Muir, *Fife Coal Company Limited*, 80.

127 MFGB, *Annual Volume of Proceedings*, 1929–30, 928.

128 NUSM, *Executive Committee*, 19 Jun. 1939, 7.

129 *Ibid*.; *Annual Conference Minutes*, 2–4 May 1940, 11.

130 NUSM, *Annual Conference Minutes*, 2–4 May 1940, 3–8.

131 NUSM, *Executive Committee Minutes*, 12 Aug. 1940, 3; 12 Oct. 1940, 6–7.

132 Bourke, *Dismembering the Male*, 48. See also Ben Curtis and Steven Thompson, ‘“A Plentiful Crop of Cripples Made by All This Progress”: Disability, artificial limbs and working-class mutualism in the South Wales coalfield, 1890–1948’, *Social History of Medicine* 27 (2014) 708–727.

133 *Colliery Guardian*, 27 Aug. 1926, 4.

134 *Colliery Guardian*, 11 Oct. 1935, 5.

135 *Colliery Guardian*, 2 Aug. 1935, 3.

136 *Colliery Guardian*, 9 May 1930, 3.

137 Hywel Francis and David Smith, *The Fed: A history of the South Wales miners in the twentieth century* (London, 1980), 439.

138 See for example McIvor and Johnston, *Miners*' *Lung*; Bloor, ‘No longer dying’; Melling and Bufton, ‘A mere matter of rock’; Ben Curtis and Steven Thompson, ‘ “This is the country of premature old men”: Ageing and aged miners in the South Wales coalfield, *c*.1880–1947’, *Cultural and Social History* 12 (2015) 587–606.

139 S. W. Fisher in *Silicosis. Records of the International Conference held at Johannesburg, 13–27 Aug. 1930*, Geneva, ILO, 1930, 83 [online http://www.ugr.es/∼amenende/investigacion/ILO-Silicosis-Conference-1930.pdf; accessed 21 March 2015].

140 Moses, ‘Contesting Risk’, 73.

141 For a wider, comparative discussion see Kirsti Bohata, Alexandra Jones, Mike Mantin and Steven Thompson, *Disability in Industrial Britain: A Cultural History of Illness, Injury and Impairment in the Coal Industry, 1880–1948* (Manchester, forthcoming).

